# From Rub Tree Prediction to Targeted Genetic Sampling in Brown Bears: Linking Scent-Marking Ecology and Spatial Modelling

**DOI:** 10.3390/life16071045

**Published:** 2026-06-23

**Authors:** Ján Barilla, Richard Hančinský, Matej Ferenčík, Jaroslav Solár, Daniel Mihálik, Ján Kraic

**Affiliations:** 1 Faculty of Natural Sciences, University of Ss. Cyril and Methodius in Trnava, Nám. J. Herdu 2, 91701 Trnava, Slovakia; barilla1@ucm.sk (J.B.); richard.hancinsky@ucm.sk (R.H.); or daniel.mihalik@nppc.sk (D.M.); 2 Faculty of Forestry and Wood Sciences, Czech University of Life Sciences Prague, Kamýcká 129, 16500 Praha 6–Suchdol, Czech Republic; ferencikmatej@fld.czu.cz; 3 Institute of High Mountain Biology, University of Žilina, Tatranská Javorina 7, 05956 Žilina, Slovakia; solarj@uniza.sk; 4 Research Institute of Plant Production, National Agricultural and Food Centre, Bratislavská cesta 122, 92168 Piešťany, Slovakia

**Keywords:** *Ursus arctos*, High Tatras, rub tree, spatial modelling, landscape-scale analysis, non-invasive genetic sampling

## Abstract

Scent marking has been discussed as an important component of communication in brown bears (*Ursus arctos* Linnaeus, 1758). However, the environmental factors influencing the occurrence of rub trees and their value for non-invasive genetic sampling remain poorly understood. This study examined the patterns of rub tree occurrence in the eastern High Tatra Mountains (Slovakia) at two spatial scales. At the tree scale, paired-design generalized linear mixed models showed that rub trees were more frequently recorded on large-diameter coniferous trees, indicating an association with visually prominent and chemically suitable substrates. At the landscape scale, logistic regression models revealed that the probability of rub tree occurrence increased with elevation and distance from human settlements, identifying high-elevation forests as areas of higher predicted rub tree occurrence. The best-supported model was used to produce a predictive map of rub tree occurrence across the study area. We also evaluated whether rub trees are reliable sources of biological material for non-invasive sampling. Hair collected during repeated field visits provided DNA suitable for genotyping and individual identification. Overall, the results show that rub trees exhibit non-random spatial patterns and represent effective focal points for systematic genetic sampling, linking patterns of rub tree occurrence to the spatial targeting of non-invasive genetic sampling in mountain landscapes.

## 1. Introduction

Understanding animal social behaviour is fundamental to ecological research and effective wildlife management [1,2], particularly when interpreted through the framework of landscape-mediated communication, where large carnivores place signals in space, which may affect their transmission and detection [3]. To convey information about individual identity [4], reproductive status, territorial boundaries, and social hierarchies to conspecifics, animals deposit chemical signals in their environment using secretions from specialized glands [5,6]. These chemical signals can persist for an extended period, enabling asynchronous communication between individuals [3]. Scent marking has been described as spatially structured, with animals using specific locations and substrates, such as tree trunks, to enhance the detectability and persistence of chemical signals [7]. This behaviour may reduce energetic costs by increasing the likelihood that signals are encountered by potential receivers [8]. These patterns indicate that scent marking is non-random and spatially structured, with animals using specific locations and substrates, which may affect signal persistence and detectability.

The marking of prominent objects, such as trees and rocks, is widespread among mammals and is particularly well-documented in carnivores [9,10]. In addition, certain substrates are more suitable for retaining chemical signals for extended periods. Eurasian lynx (*Lynx lynx* Linnaeus, 1758) do not scent mark randomly; instead, they are frequently recorded on vertical structures, such as tree trunks and stumps, which are frequently located along straight forest paths where detection by other individuals is more likely [11]. Similarly, Eurasian badgers (*Meles meles* Linnaeus, 1758) commonly establish communal latrines along territorial boundaries to delineate borders and reduce conflict with neighbouring individuals and social groups [12]. Across taxa, these examples consistently demonstrate that scent-marking behavior has been associated with non-random substrate use and spatial positioning.

Ursids, including the brown bear (*Ursus arctos* Linnaeus, 1758), have been reported to use scent marking in a communication context, have been suggested to convey information about individual identity, sex, dominance status, and reproductive condition [13,14,15]. Brown bears rely on several specialized glands to produce chemical signals, including sebaceous glands in the skin of the back [16], anal glands that produce secretions independent of feces [14], and pedal glands used for marking trails [17]. These secretions provide persistent chemical cues that enable asynchronous interactions among individuals, thereby reducing the need for direct encounters in their largely solitary lifestyle [18]. Simultaneously, they may also be associated with reproductive interactions.

Brown bears commonly use rub trees, which have been discussed in the context of chemical communication [19,20,21]. This behaviour involves clawing, biting, urinating, and rubbing various parts of the body against tree trunks, leaving both chemical and, in some cases, visual cues [22,23]. Although the functional significance of rub trees remains an active area of research, several hypotheses suggest that this behaviour may serve roles ranging from social communication and signalling to ectoparasite removal [15,19,24]. Rub trees are typically large-diameter trees commonly located along travel routes, which may increase the likelihood that scent marks will be detected by conspecifics, as documented in the American black bear (*Ursus americanus* Pallas, 1780) [21]. Consequently, rub trees may represent locations of repeated use by multiple individuals within bear populations and can be revisited by individuals of different sexes and age classes over multiple years [20]. Because of this repeated use, rub trees represent an important source of non-invasive hair samples, which are widely used in genetic studies to assess population dynamics, genetic diversity, and connectivity [25,26]. In addition, non-invasive samples such as hair or feces collected from marking sites have been used in hormonal and physiological research, providing evidence that rubbing and scent-marking behaviours are hormonally regulated and have been discussed in relation to chemically mediated communication, particularly in relation to reproductive and social signalling [17].

Scent-marking behaviour in brown bears provides valuable opportunities for non-invasive genetic analysis. Bears frequently leave hair on the substrate while rubbing against trees or other vertical structures, making these objects suitable for hair-trap sampling. Consequently, marking objects equipped with barbed wire have become widely used tools for collecting hair in population studies [26,27,28], allowing researchers to obtain high-quality genetic samples using non-invasive methods. Because rub trees are repeatedly used by multiple individuals and often retain hair suitable for genetic analyses [20,26], understanding the environmental factors shaping their occurrence has implications beyond behavioural ecology. Identifying areas where rub trees are more likely to occur can provide an important ecological context for planning and evaluating genetic sampling efforts in mountainous landscapes such as the High Tatras, thereby linking behavioural ecology with non-invasive sampling. Our study focuses on environmental correlates rather than behavioural mechanisms.

We aimed to improve the spatial targeting of non-invasive genetic sampling. To address this knowledge gap, this study aimed to answer the following questions in the Western Carpathians: (i) Are rub trees more frequently recorded on coniferous trees than on deciduous trees?

(ii) Does the probability of a tree being recorded as a rub tree increase with tree circumference?

(iii) How is the occurrence of rub trees associated with landscape-scale environmental variables, including habitat characteristics, topography, and human-related factors?

(iv) Do hair samples collected from rub trees provide DNA of sufficient quantity and quality for genetic analyses?

(v) Can the spatial distribution of rub trees be used to support the targeting of non-invasive genetic sampling?

## 2. Materials and Methods

### 2.1. Study Area

**Figure 1:**
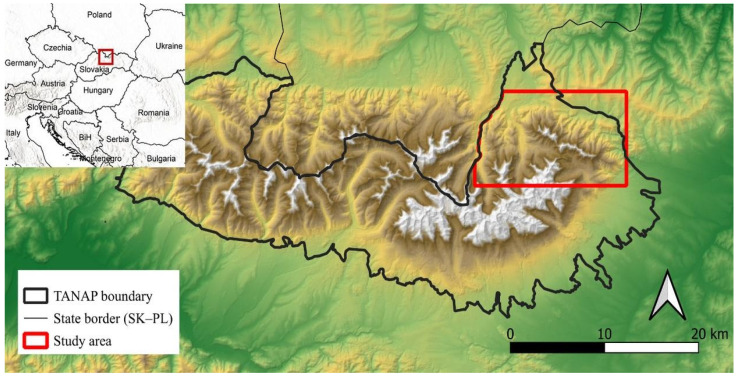
The geographic location of the study area in the eastern High Tatra Mountains and its position within Europe.

The study area was in the eastern part of the High Tatra Mountains in northern Slovakia (Figure 1) and covered approximately 150 km^2^ centered on coordinates 49°14′ N and 20°13′ E. The terrain is characterized by sharp peaks, steep slopes, and glacial valleys, with elevations ranging from 756 to 2479 m above sea level (a.s.l.).

Climatic conditions vary considerably across the study area, from the valley environment of the village of Tatranská Javorina (1000 m a.s.l.) to the alpine zone of Lomnický štít (2634 m a.s.l.). The mean annual air temperature decreases from approximately 4–5 °C at Tatranská Javorina to −3 °C at Lomnický štít, while mean annual precipitation increases from 1360 mm to >1700 mm [29]. The landscape spans two major biomes: the temperate forest biome at lower elevations and the alpine biome above the tree line (around 1550 m a.s.l.). The area is considered a biodiversity hotspot within the European temperate zone, harbouring numerous endemic species and supporting large populations of brown bear, Eurasian lynx, and wolf (*Canis lupus* Linnaeus, 1758) [30]. Temperate forests in the region are predominantly composed of coniferous species, including Norway spruce (*Picea abies* L. Carst.), European silver fir (*Abies alba* Mill.), European larch (*Larix decidua* Mill.), and Swiss stone pine (*Pinus cembra* L.), depending on local site conditions. Deciduous species include European beech (*Fagus sylvatica* L.), sycamore maple (*Acer pseudoplatanus* L.), rowan (*Sorbus aucuparia* L.), and silver birch (*Betula pendula* Roth.). The underlying geology consists primarily of granitoids, limestones, and dolomites, with leptosols and podzols predominating the soil cover.

The understory at lower elevations is rich in berry-producing shrubs, particularly species of the genus *Vaccinium*, whereas mosses and grasses primarily characterize higher elevations. Subalpine and alpine meadows dominated by dwarf mountain pine (*Pinus mugo* Turra) occur at high elevations. Forests cover approximately 71% of the study area. The area is partially protected within Tatra National Park, which provides formal protection to approximately 41.6% of the study area. Linear features, including forestry roads and hiking trails, occur at a density of approximately 0.50 km/km^−2^ across the landscape. The High Tatra brown bear population is transboundary, shared between Slovakia and Poland. Recent genetic monitoring in the Polish Tatra Mountains estimated approximately 55 individuals (95% CI: 45–79), indicating a relatively high local abundance within this mountain range [31]. In Slovakia, brown bears are typically active from April to November, with activity peaking during the hyperphagic period in late summer and autumn. Denning typically begins in November and continues until March [32]. Accordingly, field research was conducted during the bears’ active season, from April to November in 2021–2022.

### 2.2. Rub Trees

**Figure 2:**
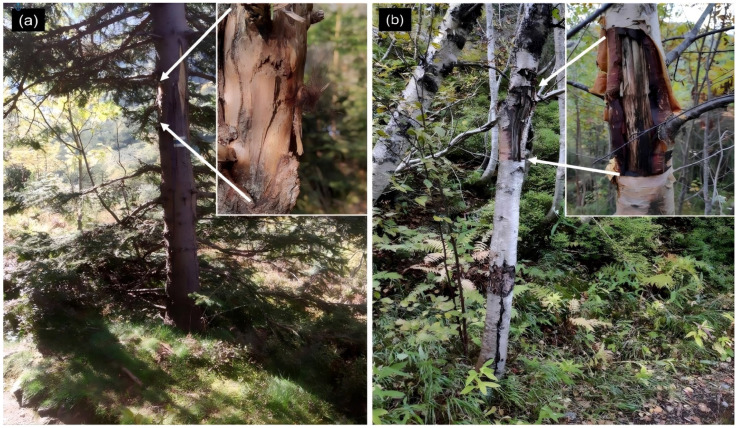
Examples of trees recorded as rub trees during field surveys. (**a**) Coniferous rub tree showing characteristic bark damage caused by rubbing behaviour, with an inset detail of hair remains used for non-invasive genetic sampling. (**b**) Deciduous rub tree exhibiting bark damage consistent with rubbing.

A rub tree was defined as a tree exhibiting characteristic signs of bear rubbing, such as smoothed or discolored bark, scratches, bites, or reduced vegetation at the base, and was only classified as a rub tree when the presence of bear hair on the trunk was confirmed. A systematic sampling design was applied to generate random points within forested areas based on the forest inventory layer provided by the National Forest Center (NFC, Zvolen, Slovakia). At each point, the nearest tree was identified and recorded as a reference (absence point), and a standardized search for rub trees was conducted within a circular plot with a radius of 30 m. No rub trees were detected within these plots. To obtain sufficient presence records, we additionally documented opportunistically encountered rub trees while accessing the randomly generated sampling points. Subsequently, two datasets were constructed to investigate rub tree occurrence at different spatial scales. Figure 2 shows an example of a tree classified as a rub tree in this study, illustrating typical signs of bear rubbing.

### 2.3. Tree-Scale Analysis of Rub Tree Occurrence

The tree-scale patterns of rub tree occurrence were evaluated using a paired-design generalized linear mixed model (GLMM). Field surveys identified 78 matched tree pairs (one rub tree and one nearby random tree within ~30 m), yielding a total of 156 observations. For each tree, taxonomic identity (grouped as coniferous or deciduous) and circumference at breast height (CBH, cm), measured 1.3 m above the ground, were recorded. Tree types were coded as a two-level categorical variable (coniferous/deciduous), and CBH was standardized to a mean of 0 and a standard deviation of 1 (CBH_std). The response variable indicated whether a tree was recorded as a rub tree (1 = rub tree, 0 = paired random tree). Pair identity (ID) was included as a random intercept to account for the matched-pair design. The model was fitted using a binomial error distribution with the following logit link function:

logit[P(rub tree = 1)] = β_0_ + β_1_(species) + β_2_(CBH_std) + (1|pair_ID)

where P(rub tree = 1) is the probability that a tree is recorded as a rub tree (“rub tree”); logit(P) is the logarithm of the odds of this event, defined as logit(*p*) = ln(*p*/1 − *p*), β_0_ is the intercept, representing the baseline log-odds when all predictors are equal to zero; β_1_(species) is the coefficient for the species variable (tree type, e.g., coniferous vs. deciduous) and indicates how the log-odds of a tree being recorded as a rub tree change with species; β_2_(CBH_std) is the coefficient for standardized circumference at breast height (CBH), describing how tree size is associated with the log-odds of a tree being recorded as a rub tree; (1|pair_ID) represents the random intercept for pair_ID (e.g., matched tree pairs), accounting for unobserved heterogeneity among pairs and capturing the correlation between trees within the same pair.

All analyses were conducted in the R environment (version 4.3.0) [33]. Data cleaning and preparation were performed using the dplyr v. 1.2.1 (available online: https://cran.r-project.org/web/packages/dplyr/index.html, accessed on 18 March 2026) and readr v. 2.2.0 (available online: https://cran.r-project.org/web/packages/readr/index.html, accessed on 18 March 2026) packages. The model was fitted using lme4::glmer (using R-4.5.3 for Windows available online: https://cran.r-project.org/, accessed on 18 March 2026 and RStudio v. 2026.01.2+418 available online: https://posit.co/download/rstudio-desktop, accessed on 27 March 2026) with the bobyqa optimizer, and the maximum number of function evaluations maxfun was set to 2 × 10^5^ to ensure model convergence. Fixed-effect estimates are reported as odds ratios (OR) with Wald 95% confidence intervals and *p*-values. Model performance was summarized using the Akaike Information Criterion (AIC) and Nakagawa’s marginal and conditional R^2^ calculated with the performance package v 0.16.0 (available online: https://cran.r-project.org/web/packages/performance/index.html, accessed on 18 March 2026). Model assumptions were evaluated using DHARMa v 0.4.7 (tests for uniformity, dispersion, and outliers, available online: https://cran.r-project.org/web/packages/DHARMa/index.html, accessed on 18 March 2026). Multicollinearity among predictors was assessed using performance::check_collinearity (variance inflation factors), and the influence of individual tree pairs was evaluated with the influence.ME package v. 0.9-10 (Cook’s distance, available online: https://cran.r-project.org/web/packages/influence.ME/index.html, accessed on 18 March 2026). In contrast to the landscape-scale analysis, these variables were obtained directly from field measurements, with each rub tree paired with a nearby random tree (within ~30 m) to control for local environmental conditions.

### 2.4. Landscape-Scale Analysis of Rub Tree Occurrence

To evaluate landscape-scale predictors of rub tree occurrence in the eastern High Tatras, we constructed a presence–absence dataset comprising 78 field-verified rub trees and 78 randomly selected forest points. The presence and absence were coded as a binary variable (1 = rub tree, 0 = random tree). Eight GIS-derived environmental predictors were used to describe habitat, topography, and human impact at a spatial resolution of 25 m. These included vegetation greenness (NDVI), forest age (FAGE), elevation, slope, terrain ruggedness (TRI), topographic position (TPI), and distances to roads and buildings (see Appendix A for detailed definitions and processing).

- Habitat: FAGE and NDVI.
- Topography: elevation, slope, TRI, and TPI.
- Human impact: distance to roads and distance to buildings.

All raster layers were processed using QGIS (version 3.28.13) [34] and R (version 4.3.0) with the terra and sf packages, respectively. Raster-derived predictor variables were reprojected to the S-JTSK/Krovak East North coordinate system [35], aligned to a 25 m grid, and masked to forest cover based on the forest inventory layer (a geospatial dataset of forest stands and their attributes provided by the National Forest Centre). All continuous predictors were standardized (mean = 0, standard deviation = 1) before analysis. All predictors were extracted at each presence and random point and used as explanatory variables in subsequent logistic regression models.

### 2.5. Statistical Analysis

Logistic regression (generalized linear model, binomial family with a logit link) was used to model the occurrence of rub trees as a function of environmental predictors. The generalized linear models were specified in the following form:

logit[P(rub tree = 1)] = β_0_ + β_1_(X_1_) + β_2_(X_2_) + β_3_(X_3_)

where P denotes the probability of rub tree occurrence, β_0_ is the intercept, representing the log-odds of a tree being recorded as a rub tree when all predictors (X_1_, X_2_, and X_3_) equal zero, and β_1_, β_2_, and β_3_ are regression coefficients representing the effects of habitat, topographic, and human-impact predictors included in each candidate model, respectively. We tested all three-predictor models that included one variable from each ecological domain, yielding 16 candidate models. Models were ranked using the Akaike Information Criterion (AIC), the area under the receiver operating characteristic curve (AUC) [36], and Tjur’s R^2^ [37]. All analyses were conducted in R 4.3.0 [33] using the pROC v. 1.19.0.1 (available online: https://cran.r-project.org/web/packages/pROC/index.html, accessed on 18 March 2026) [38], performance [39], and DHARMa [40] packages, version 0.4.6 (available on 18 March 2026). Diagnostic evaluation of the top-ranked model included residual tests implemented in DHARMa and assessment of multicollinearity using the check_collinearity function from the performance package.

#### 2.5.1. Model Evaluation and Prediction Map

Model performance was assessed using the area under the receiver operating characteristic curve (AUC) to measure discrimination and Tjur’s R^2^ (the difference between the mean predicted probabilities for positive and negative cases) as an additional measure of explained variance. Model fit and residual diagnostics were evaluated using the DHARMa package through tests for uniformity, overdispersion, and outliers. Multicollinearity among predictors was assessed using the check_collinearity function from the performance package (variance inflation factors). The best-supported GLM—the top-ranked three-predictor model containing one variable from each ecological domain (habitat, topography, and human impact)—was then applied across the study area to generate a raster of predicted rub tree occurrence probabilities (0–1). All predictions were produced in R (version 4.3.0) using the terra package [41] and masked to forest pixels using the forest age variable derived from the forest stand map (NFC), thereby restricting the prediction map to suitable habitat.

#### 2.5.2. Non-Invasive Genetic Sampling

Non-invasive genetic sampling conducted in 2022 was independent of the ecological data used to model the occurrence of the rub tree. Transect surveys of approximately comparable length were conducted monthly within each 5 × 5 km grid cell. During these surveys, transects were walked to search for signs of bears and potential sources of non-invasive genetic material, and samples were collected opportunistically. To reduce the risk of mixing hair from different individuals, hair samples consisting of more than 10 hairs with follicles were collected from a limited portion of the rubbed tree surface and stored dry in paper envelopes for further analysis. Remaining hairs from the same tree were subsequently collected separately as mixed samples and stored in additional envelopes; however, these mixed samples were not included in genetic analyses. After sampling, the rubbed surface was cleared of hair to minimize the likelihood of repeated sampling during subsequent visits. Fresh feces were collected opportunistically during field surveys and preserved in ethanol. Their age was estimated to be no more than five days based on field assessment of characteristics such as moisture, color, and degree of decomposition. However, due to the low number of fecal samples collected (*n* = 5), they were not included in subsequent genetic analyses. DNA was extracted from the hair samples following established protocols for genetic analyses of brown bears [42]. Extractions were performed using the Qiagen DNeasy Blood & Tissue Kit (Qiagen N.V., Hilden, Germany). DNA concentration (ng/µL) and purity (A_260_/A_280_ and A_260_/A_230_) were measured spectrophotometrically using a NanoDrop One Spectrophotometer (ThermoFisher Scientific Inc., Waltham, MA, USA) based on A260/A280 and A260/A230 ratios (concentration expressed in ng/µL). Genetic analyses were conducted using six microsatellite loci selected from the European brown bear microsatellite panel, along with the sex-specific SRY marker (Table 1).

Loci were amplified using multiplex polymerase chain reaction (PCR) with the QIAGEN Multiplex PCR Kit (Qiagen N.V., Hilden, Germany). PCR amplification was performed with an initial denaturation at 95 °C for 15 min, followed by 38 cycles of 95 °C for 1 min, 58 °C for 1 min 30 s, and 72 °C for 1 min, with a final extension at 60 °C for 30 min using the GeneAmp PCR System 9700 (Applied Biosystems, Thermo Fisher Scientific Inc., Waltham, MA, USA). Fragment analysis was performed using the Applied Biosystems SeqStudio Genetic Analyzer System (Thermo Fisher Scientific Inc., Waltham, MA, USA). Data were processed using the SeqStudio Analysis Software, version 1.1 (Thermo Fisher Scientific Inc., Waltham, MA, USA), including GeneMapper™ Software 6, version 6.1 (Thermo Fisher Scientific Inc., Waltham, MA, USA), for allele sizing. The genetic parameters evaluated included the number of alleles per locus (N_a_), observed heterozygosity (H_o_), expected heterozygosity (H_e_), and allelic richness (Ar). These parameters were calculated using FSTAT (version 1.2) [47] to estimate genetic diversity indices.

## 3. Results

### 3.1. Tree-Scale Patterns of Rub Tree Occurrence

Deciduous trees had significantly lower odds of being recorded as rub trees compared with conifers (Table 2), indicating a higher frequency of rub trees on coniferous trees. Tree size showed a positive association with rub tree occurrence, with larger trees more likely to be recorded as rub trees. The model showed acceptable fit (Table 2), and diagnostic checks indicated no violation of model assumptions. Descriptive statistics for the tree-scale dataset showed that rub trees were predominantly coniferous (76 of 78), whereas paired random trees included a higher proportion of deciduous trees (15 of 78). Mean CBH was higher for rub trees (86.9 ± 35.4 cm) than for paired random trees (64.0 ± 46.1 cm).

### 3.2. Landscape-Scale Predictors of Rub Tree Occurrence

Model selection identified a best-supported model including NDVI, elevation, and distance to buildings (Appendix B). Elevation showed a positive association with rub tree occurrence, indicating higher predicted occurrence at higher elevations. In contrast, distance to buildings was negatively associated with rub tree occurrence, indicating higher predicted occurrence in areas farther from human infrastructure. NDVI exhibited a weak and non-significant effect (Table 3).

Several competing models with similar support consistently included elevation and distance to buildings, highlighting these variables as being associated with rub tree occurrence, whereas vegetation-related predictors varied among models (Appendix B).

Model diagnostics indicated no violations of assumptions, with residuals showing no evidence of overdispersion or outliers. Multicollinearity was low across predictors, confirming the robustness of the model results (Table 4).

### 3.3. Spatial Prediction of Rub Tree Occurrence

**Figure 3:**
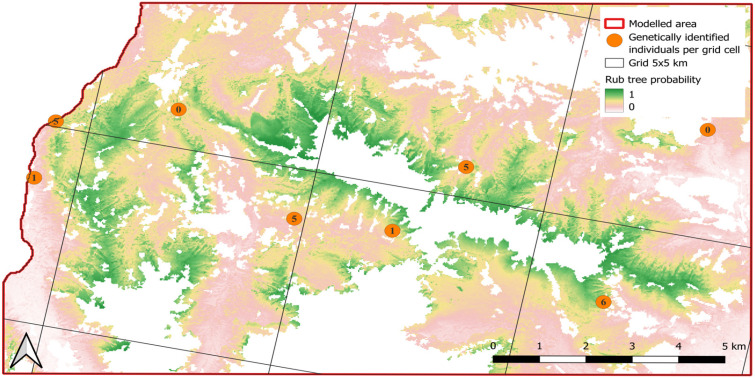
Predicted probability (0–1) of rub tree occurrence based on the best-supported generalized linear model (GLM), including elevation, distance to buildings, and NDVI. The colour scale represents predicted probability from low (white) to high (green). Orange points indicate surveyed 5 × 5 km grid cells repeatedly visited for non-invasive genetic sampling, with numbers indicating the number of genetically identified individuals within each grid cell.

The best-supported GLM was applied across the study area to generate a spatial probability map of rub tree occurrence (Figure 3). Predicted probabilities were highest in mid- to high-elevation forest areas located farther from buildings, consistent with the significant model predictors. The spatial prediction revealed a heterogeneous distribution of rub tree occurrence, with distinct clusters of probability areas indicating areas of higher predicted occurrence within the landscape. These patterns indicate that the distribution of rub trees is non-random across the landscape. Predictions were restricted to forested habitats using the forest-age layer from the NFC dataset.

### 3.4. Non-Invasive Genetic Sampling from Rub Trees

The predicted probability values extracted at the rub tree locations where the hair samples were collected (n = 31) ranged from 0.147 to 0.757, with a mean of 0.464 ± 0.180. Multiple samples were collected from the same rub trees; therefore, some samples shared identical predicted probability values. Overall, these results indicate that most hair samples were obtained from areas with moderate to high predicted probability of rub tree occurrence.

A total of 36 non-invasive samples were collected during regular field surveys, primarily hair samples from rub trees. DNA quality and quantity varied among samples. DNA suitable for subsequent molecular analyses could not be obtained from eight hair samples. In the remaining 23 samples, DNA concentration was low (ng/µL range), and DNA quality was suboptimal, with mean A260/A280 and A260/A230 ratios of 1.70 and 0.74, respectively. Consequently, genotyping using six microsatellite markers was performed for 74.2% of the collected hair samples. Rub trees facilitated the non-invasive collection of biological material from brown bears, although DNA could not be recovered from all samples. Despite lower quantity and quality, the isolated DNA was sufficient for individual identification and genotyping using multilocus microsatellite analysis. From 23 samples, 20 unique brown bear individuals were identified. Sex determination using the SRY marker revealed 13 males and 10 females. Overall, these results demonstrate that rub trees can serve as effective focal points for non-invasive genetic sampling, particularly when sampling is guided by spatial predictions of marking-site occurrence.

Genetic diversity across the analysed loci was generally high, with multiple alleles per locus and consistently elevated heterozygosity values. No significant deviations from the Hardy–Weinberg equilibrium were detected after Bonferroni correction, suggesting that the analysed loci reflect stable population genetic structure without strong effects of inbreeding or selection. These findings confirm that the selected microsatellite markers provide a reliable basis for assessing genetic diversity using non-invasive samples collected from rub trees (Table 5).

## 4. Discussion

This study provides a multi-scale analysis of scent-marking behavior in brown bears and presents the first explicit prediction of rub tree distribution in the High Tatra Mountains. By integrating tree-scale patterns of rub tree occurrence, landscape-scale modelling, and targeted non-invasive genetic sampling, we show that rub tree occurrence is non-randomly distributed and forms predictable spatial patterns across the landscape.

The higher frequency of rub trees on coniferous species aligns with previous research indicating that resinous species are commonly used as rub trees, as their bark structure and chemical composition may allow chemical signals to persist for longer periods [1,11,48]. Conifers, such as spruce and larch, also possess rough bark and are visually prominent along travel routes, which may increase the likelihood that scent marks are detected by conspecifics [20,49]. The positive association with tree circumference is consistent with findings from other regions [21,50], suggesting that large trees may function as stable marking objects that accumulate scent cues and hairs through repeated use. These findings indicate that physical tree characteristics may play a role in the occurrence of marking sites, potentially influencing the persistence and detectability of chemical signals [11,15].

At the landscape scale, the probability of rub tree occurrence increased with elevation, indicating higher predicted occurrence at higher elevations. These patterns are consistent with higher frequencies of rub trees in high-elevation forests. Elevation has been reported to be associated with variation in rub tree occurrence in large, continuous wilderness areas such as Yellowstone, where rub trees are more frequently recorded at lower elevations and where forests remain relatively undisturbed, and suitable marking substrates are abundant [21]. In contrast, studies from European mountain systems reported no significant elevation effects, although there were tendencies for higher frequencies of rub trees at higher elevations, often corresponding to less fragmented landscapes with lower road density, reduced settlement pressure, and limited agricultural activity [49,51,52]. Furthermore, human-modified landscapes, combined with limited marking substrates above the treeline and intensive tourism with dense trail networks at mid-elevations, may obscure or weaken elevation-related patterns. In the High Tatra Mountains, both increasing elevation and greater distance from buildings were associated with a higher probability of rub tree occurrence, consistent with higher frequencies of rub trees in high-elevation forests with lower human pressure and greater forest continuity. A similar pattern was reported in the Italian Alps, where rub tree frequency declined near settlements and paved roads [52].

A study from northwestern Montana, USA, found that rub tree locations were unrelated to NDVI, which was excluded from all supported models [53]. In our study, NDVI showed a weak, non-significant effect, suggesting that rub trees were slightly less likely to occur in areas with very high vegetation greenness. Because random points in our analysis were sampled only within forested areas, NDVI likely reflected variation in canopy density rather than a contrast between forested and non-forested habitats. Across other studies, rub tree placement often aligns with locally open structures, such as along paths or roads and on visually conspicuous trees, and may be associated with animal movement and signal detectability [49,52].

Our results indicate that rub tree occurrence in the High Tatras reflects a trade-off between spatial patterns of rub tree occurrence and risk avoidance [22,54]. Brown bears’ rub trees were more frequently recorded on large coniferous trees in structurally complex, high-elevation forests with minimal human disturbance, a pattern similar to that of other solitary carnivores, such as felids, which maintain indirect social networks through scent marking and spatially structured interactions [55,56]. These areas may represent locations of repeated use along travel corridors, enabling individuals to exchange asynchronous information [54]. The observed elevation pattern may also reflect seasonal habitat shifts, as the use of resting and movement sites may vary during the late summer and autumn hyperphagia periods [51]. Overall, these findings are consistent with the view that scent marking has been discussed as a form of chemical communication in solitary carnivores, allowing individuals to advertise their presence while minimizing the need for direct encounters [57]. Together, these results suggest that rub tree occurrence is associated with topographic and human-related factors rather than vegetation structure.

Rub tree occurrence appears to be structured by consistent environmental gradients, suggesting that the distribution of rub trees is non-random and reflects broader landscape patterns [58,59]. Although the model is not intended to predict individual marking events, the resulting probability map identifies high-elevation forests in the Tatra Mountains, located farther from occupied buildings, as potential rub tree hotspots. Such spatial predictions can inform targeted field surveys in the Western Carpathians, optimize the placement of hair traps commonly used for non-invasive genetic sampling, and enhance overall sampling efficiency [25,26,28]. Importantly, this represents one of the first attempts to spatially predict marking-site distribution in a European bear population.

Recent predictive modelling approaches have been applied to map habitat suitability and behavioural hotspots in large carnivores [60,61]. Such spatial models can identify environmental and anthropogenic gradients that are associated with rub tree occurrence and guide the design of non-invasive genetic sampling. Integrating predictive models of rub tree distribution into long-term sampling frameworks could also help delineate movement corridors and areas of repeated use that are critical for maintaining population connectivity [62].

Beyond its ecological interpretation, the recovery of individual genotypes from non-invasive samples collected at systematically surveyed rub trees illustrates how spatial modelling can support the identification of rub tree occurrence patterns and inform fieldwork decisions at both the landscape and tree scales. Specifically, the model functioned as a practical tool for directing searches toward suitable areas at the landscape scale and for identifying appropriate marking trees within those areas when selecting a single sampling tree within 5 × 5 km grid cells, thereby facilitating efficient long-term genetic sampling under realistic field conditions. This analysis relied on field data and GIS-derived environmental layers; consequently, it did not capture seasonal variation in marking intensity or temporal changes in human activity [63]. Rub tree data included both systematically and opportunistically collected points, which may introduce spatial bias [64]. Future studies should incorporate dynamic predictors, such as seasonal movements derived from GPS collars or human visitation intensity (e.g., Strava-based data), to better capture temporal variation [65]. Independent datasets from adjacent, similarly human-influenced regions, such as the Western and Low Tatras, could be used to test model transferability. Evaluating performance on independent data is essential for assessing predictive robustness and ecological generality, as simple, parsimonious models applied within environmentally similar regions often achieve higher transfer success [66,67]. The systematically collected 5 × 5 km rub tree dataset provides an ecological framework for future presence-only modelling approaches (e.g., MaxEnt), which can be applied at broader spatial scales to complement the current presence–absence framework. This approach may also be particularly valuable for transboundary populations, such as those in the Carpathian region, where coordinated monitoring across national borders remains challenging. Spatially explicit predictions of areas of higher predicted rub tree occurrence provide a standardized framework that can be applied across regions, facilitating harmonized sampling strategies and improving comparability of genetic data.

Although genetic analysis was not the primary focus of this study, it provided an important validation of the ecological and spatial predictions. Non-invasive genetic sampling using hair collected from rub trees has become a widely applied method for monitoring brown bear populations. However, DNA extraction from hair presents several well-recognized methodological challenges. A key limitation of non-invasive sampling is the low quantity and often degraded state of DNA, which increases the likelihood of amplification failure and genotyping errors, such as allelic dropout and false alleles [68]. Successful DNA recovery strongly depends on the presence of intact hair follicles, whereas shed guard hair without roots frequently contains insufficient nuclear DNA for reliable genotyping. Environmental exposure before collection further reduces DNA quantity, as prolonged sunlight, humidity, and microbial activity accelerate DNA degradation [69]. Previous brown bear monitoring studies report that a proportion of hair samples fail due to low DNA quality or contamination, highlighting the inherent uncertainty of field-collected material [70]. The success rate of DNA recovery from opportunistically collected hair samples in the field is often very low, around 15% [46]. Additionally, repeated use of rubbing sites by multiple individuals can produce mixed DNA samples, necessitating strict laboratory protocols and replication strategies to minimize identification errors [71]. Empirical studies also show that substantial variation in genotyping success depends on sampling strategy and sample type. In a large-scale genetic monitoring study of brown bears in Slovakia, overall genotyping success for non-invasive samples reached approximately 46%, with 47% for fecal samples and only 24% for hair samples collected opportunistically from rub trees [72]. The low success rate of hair samples was primarily attributed to poor sample quality and the limited number of hair follicles available for DNA extraction. In contrast, in our study, hair samples collected from systematically monitored rub trees during monthly field surveys achieved a genotyping success rate of 74.2%, suggesting that targeted sampling at frequently used marking sites may substantially increase the efficiency of non-invasive genetic monitoring. Despite inherent constraints, optimized sampling strategies, rapid sample preservation, and multiple-tube PCR approaches can markedly improve genotyping success, confirming that rub-tree hair sampling remains an effective and minimally invasive tool for large carnivore monitoring and the assessment of conservation genetics [46]. The relatively high observed heterozygosity indicates that individuals using rub trees represent a genetically diverse subset of the local population, rather than a limited social or kin-based group. Combining behavioural ecology with non-invasive genetics provides significant advantages for conservation monitoring. Rub-tree sampling allows repeated detection of individuals across seasons and years without capture or disturbance, facilitating long-term monitoring of population size, connectivity, and genetic health [25,73]. In the Carpathian region, where brown bears inhabit increasingly human-modified landscapes, such approaches provide essential data for evaluating population viability, identifying dispersal corridors, and assessing potential fragmentation risks.

Overall, our results demonstrate that rub trees can serve as natural sampling points associated with spatial ecology and genetic monitoring. By providing the first spatially explicit prediction of rub tree occurrence in this region and linking these predictions with genetic validation, this study offers a novel framework for improving non-invasive sampling strategies. This integrative approach highlights the potential of spatial models informed by observed patterns to enhance wildlife monitoring, support conservation planning, and contribute to the management of large carnivore populations in human-influenced and transboundary mountain landscapes.

## 5. Conclusions

This study demonstrates that the occurrence of rub trees in the eastern High Tatra Mountains is associated with a combination of fine-scale tree characteristics and broader landscape-scale factors related to topography and human disturbance. At the tree scale, bears were more frequently recorded on large-diameter coniferous trees, highlighting the importance of visually prominent and chemically suitable substrates for effective scent communication. At the landscape scale, the occurrence of rub trees increased with elevation and distance from buildings, indicating higher predicted occurrence in remote, high-elevation forests with lower human pressure. By integrating field observations with spatial modelling, this study provides the first spatially explicit prediction of rub tree distribution in the Western Carpathians. This represents a significant advancement over previous research by moving beyond descriptive analyses towards a predictive framework that identifies areas of higher predicted rub tree occurrence across the landscape. The predictive model offers a practical tool for conservation and non-invasive genetic sampling by identifying areas with a high probability of rub tree occurrence, thereby facilitating targeted behavioral surveys and improving sampling efficiency. Genetic analysis further confirmed that rub trees can serve as effective natural sampling substrates, enabling reliable individual identification and capturing genetic variation within the local population. Overall, this study demonstrates that being informed by observed patterns can bridge the gap between ecological theory and applied conservation, providing a transferable framework for improving wildlife monitoring and management in human-influenced and transboundary mountain landscapes.

## Figures and Tables

**Table 1:** Microsatellite loci, sequences, and fluorescent labels of primers used for DNA analysis of brown bears.

Locus	Primer	5′ Label (Dye)	Primer Sequence (5′→3′)
SRY ^a^	Forward	None	GAACGCATTCTTGGTGTGGTC
	Reverse	PET^®^	TGATCTCTGAGTTTTGCATTTG
G10C ^c^	Forward	VIC^®^	AAAGCAGAAGGCCTTGATTTCCTG
	Reverse	None	GGGACATAAACACCGAGACAGC
G10L ^c^	Forward	PET^®^	ACTGATTTTATTCACATTTCCC
	Reverse	None	GATACAGAAACCTACCCATGCG
Mu10 ^b^	Forward	None	ATTCAGATTTCATCAGTTTGACA
	Reverse	FAM^TM^	TCAGCATAGTTACACAAATCTCC
Mu23 ^d^	Forward	None	GCCTGTGTGCTATTTTATCC
	Reverse	NED^TM^	TAGACCACCAAGGCATCAG
Mu50 ^d^	Forward	None	GTCTCTGTCATTTCCCCATC
	Reverse	FAM^TM^	AACCTGGAACAAAAATTAACAC
Mu59 ^d^	Forward	None	GCTCCTTTGGGACATTGTAA
	Reverse	NED^TM^	TGACTGTCACCAGCAGGAG

Primers designed by ^a^ [43], ^b^ [44], ^c^ [45], and ^d^ [46].

**Table 2:** Results of the generalized linear mixed model (GLMM) evaluating tree-scale patterns of rub tree occurrence by brown bears in the eastern High Tatras. Odds ratios (OR) and corresponding *p*-values are presented. CBH std refers to the standardized circumference at breast height.

Predictors	Odds Ratio (OR)	*p*
(Intercept)	1.18	0.345
Species (deciduous)	0.13	0.011
CBH std	1.68	0.005
	Value	
σ^2^ (residual)	3.29	
τ_00_ (pair_ID)	0.00	
N (pair_ID)	78	
Observations	156	
Marginal R^2^/Conditional R^2^	0.195/NA	
AIC	203.21	

**Table 3:** The top-ranked generalized linear model (GLM) explaining the presence of rub trees in the eastern High Tatras. Odds ratios (OR) are presented with 95% confidence intervals (CI) and *p*-values.

Predictor	OR	Lower CI	Upper CI	*p*-Value
(Intercept)	5.25	0.75	36.74	0.095
NDVI	0.015	0.00012	1.79	0.085
Elevation	3.45	1.91	6.21	<0.001
Distance-to-buildings	0.41	0.23	0.71	0.002

**Table 4:** Summary of model selection for candidate three-predictor generalized linear models (GLMs) explaining rub tree occurrence in the High Tatras. Models are ranked by AIC; only models with ΔAIC < 5 are shown.

Rank	Predictors	AIC	ΔAIC	AUC
1	NDVI + Elevation + Distance to Buildings	198.7	0.0	0.74
2	Forest age + Elevation + Distance to Buildings	201.4	2.6	0.71
3	NDVI + TPI + Distance to Roads	202.4	3.7	0.71
4	NDVI + TPI + Distance to Buildings	203.4	4.7	0.71

**Table 5:** Genetic parameters obtained from DNA microsatellite analysis of non-invasive rub tree samples. The table reports the number of genotyped individuals (N), number of alleles (N_a_), observed heterozygosity (H_o_), expected heterozygosity (H_e_), and allelic richness (Ar).

Locus	N	N_a_	H_o_	H_e_	Ar
Mu50	23	6	0.76	0.64	5.92
Mu10	23	7	0.69	0.52	6.92
G10L	23	5	0.37	0.27	4.84
G10C	23	5	0.67	0.72	4.92
Mu59	23	5	0.71	0.60	4.96
Mu23	23	7	0.82	0.80	6.96

## Data Availability

The data presented in this study are available from the first author upon reasonable request. Spatial coordinates of rub tree locations are not publicly available due to conservation and species protection considerations.

## Abbreviations

The following abbreviations are used in this manuscript:

AIC	Akaike Information Criterion
a.s.l.	Above Sea Level
AUC	Area Under the Curve
CBH	Circumference at Breast Height
CI	Confidence Interval
DEM	Digital Elevation Model
DHARMa	Diagnostic Help for the Analysis of Random Mixed-Effects Models
DNA	Deoxyribonucleic acid
FAGE	Forest Age
FAM^TM^	fluorescent dye 6-carboxyfluorescein
GIS	Geographic Information System
GLM	Generalized linear model
GLMM	Generalized Linear Mixed Model
ID	Identifier
NED^TM^	fluorescent dye 2′-chloro-5′-fluoro-7′,8′-benzo-1,4-dichloro-6-carboxyfluorescein
NDVI	Normalized Difference Vegetation Index
NFC	National Forest Centre
OR	Odds ratio
OSM	OpenStreetMap
PET^®^	Red-orange, fluorescent dye originally developed by Applied Biosystems
PCR	Polymerase chain reaction
QGIS	Quantum Geographical Information System
SD	Standard deviation
TPI	Topographic Position Index
TRI	Terrain ruggedness index
VIC^®^	fluorescent dye 2′-chloro-7′-phenyl-1,4-dichloro-6-carboxyfluorescein

## Appendix A

Environmental predictor variables used in the landscape rub tree selection analysis. Each continuous variable was processed on a 25 m grid and z-standardized prior to modelling. Specific data sources and processing steps are listed below.

**Variable Name**	**Description/** **Ecological Meaning**	**Source Data**	**Processing**
NDVI	Normalized Difference Vegetation Index (vegetation greenness)	Copernicus Sentinel-2A imagery, Bands 8 and 4 (10 m resolution)	NDVI calculated as (B8 − B4)/(B8 + B4); resampled to 25 m to match analysis resolution
FAGE	Forest age (years)	Slovak National Forest Inventory (NFC)	Derived from national forest inventory; used to restrict prediction to suitable forest habitat
Elevation	Digital elevation (meters ASL)	Copernicus DEM	Base raster layer; used to align all environmental predictors to common grid
Slope	Terrain steepness (degrees)	Copernicus DEM	Calculated from DEM using standard slope algorithm
Distance to buildings	Distance to occupied buildings (m)	OpenStreetMap (OSM) occupied building layer	Euclidean distance calculated from building polygons; manual correction of OSM layer where required
Distance to roads	Distance to drivable roads (m)	OpenStreetMap (OSM) drivable roads layer	Euclidean distance calculated from road network
TRI	Terrain Ruggedness Index	Copernicus DEM	Computed from DEM using terrain ruggedness function
TPI	Topographic Position Index	Copernicus DEM	Computed from DEM using focal neighborhood analysis

## Appendix B

Competing models including elevation, distance from buildings, topographic position, terrain ruggeness, and forest age, related to the occurrence of rub trees.

**Model**	**AIC**	**AUC**	**Tjur’s R^2^**	**Predictors**	**Formula**	**Rank**
M7	198.73	0.74	0.16	NDVI + scaled_Elevation + scaled_Buildings	P_A ~ NDVI + scaled_Elevation + scaled_Buildings	1
M15	201.36	0.71	0.14	scaled_FAGE + scaled_Elevation + scaled_Buildings	P_A ~ scaled_FAGE + scaled_Elevation + scaled_Buildings	2
M2	202.43	0.71	0.13	NDVI + TPI + scaled_Roads	P_A ~ NDVI + TPI + scaled_Roads	3
M1	203.44	0.71	0.13	NDVI + TPI + scaled_Buildings	P_A ~ NDVI + TPI + scaled_Buildings	4
M8	206.09	0.69	0.11	NDVI + scaled_Elevation + scaled_Roads	P_A ~ NDVI + scaled_Elevation + scaled_Roads	5
M10	206.76	0.69	0.11	scaled_FAGE + TPI + scaled_Roads	P_A ~ scaled_FAGE + TPI + scaled_Roads	6
M3	207.04	0.68	0.10	NDVI + TRI + scaled_Buildings	P_A ~ NDVI + TRI + scaled_Buildings	7
M9	207.23	0.69	0.11	scaled_FAGE + TPI + scaled_Buildings	P_A ~ scaled_FAGE + TPI + scaled_Buildings	8
M4	208.07	0.68	0.10	NDVI + TRI + scaled_Roads	P_A ~ NDVI + TRI + scaled_Roads	9
M16	209.03	0.66	0.09	scaled_FAGE + scaled_Elevation + scaled_Roads	P_A ~ scaled_FAGE + scaled_Elevation + scaled_Roads	10
M11	212.33	0.63	0.07	scaled_FAGE + TRI + scaled_Buildings	P_A ~ scaled_FAGE + TRI + scaled_Buildings	11
M12	213.51	0.63	0.07	scaled_FAGE + TRI + scaled_Roads	P_A ~ scaled_FAGE + TRI + scaled_Roads	12
M6	217.77	0.61	0.04	NDVI + scaled_Slope + scaled_Roads	P_A ~ NDVI + scaled_Slope + scaled_Roads	13
M5	218.13	0.61	0.04	NDVI + scaled_Slope + scaled_Buildings	P_A ~ NDVI + scaled_Slope + scaled_Buildings	14
M14	222.25	0.57	0.01	scaled_FAGE + scaled_Slope + scaled_Roads	P_A ~ scaled_FAGE + scaled_Slope + scaled_Roads	15
M13	222.38	0.56	0.01	scaled_FAGE + scaled_Slope + scaled_Buildings	P_A ~ scaled_FAGE + scaled_Slope + scaled_Buildings	16
